# Quantitative genetics of plumage color: lifetime effects of early nest environment on a colorful sexual signal

**DOI:** 10.1002/ece3.1602

**Published:** 2015-07-24

**Authors:** Joanna K Hubbard, Brittany R Jenkins, Rebecca J Safran

**Affiliations:** Department of Ecology and Evolutionary Biology, University of Colorado BoulderRamaley N122, UCB 334, Boulder, Colorado, 80309

**Keywords:** Animal model, barn swallow, developmental plasticity, heritability, melanin plumage color

## Abstract

Phenotypic differences among individuals are often linked to differential survival and mating success. Quantifying the relative influence of genetic and environmental variation on phenotype allows evolutionary biologists to make predictions about the potential for a given trait to respond to selection and various aspects of environmental variation. In particular, the environment individuals experience during early development can have lasting effects on phenotype later in life. Here, we used a natural full-sib/half-sib design as well as within-individual longitudinal analyses to examine genetic and various environmental influences on plumage color. We find that variation in melanin-based plumage color – a trait known to influence mating success in adult North American barn swallows (*Hirundo rustica erythrogaster*) *–* is influenced by both genetics and aspects of the developmental environment, including variation due to the maternal phenotype and the nest environment. Within individuals, nestling color is predictive of adult color. Accordingly, these early environmental influences are relevant to the sexually selected plumage color variation in adults. Early environmental conditions appear to have important lifelong implications for individual reproductive performance through sexual signal development in barn swallows. Our results indicate that feather color variation conveys information about developmental conditions and maternal care alleles to potential mates in North American barn swallows. Melanin-based colors are used for sexual signaling in many organisms, and our study suggests that these signals may be more sensitive to environmental variation than previously thought.

## Introduction

Morphological signals including horns, antlers, and plumage ornaments are important aspects of an individual's phenotype used to attract mates and defend territories and resources necessary for reproduction (Andersson 1994). Individuals, typically males, use these sexual signals in displays or combat and these signals are consequently linked to reproductive success. An individual's phenotype is the product of both its genotype and the environment in which it is developed and expressed (Roulin and Dijkstra 2003; Garant et al. 2004; Ingleby et al. 2010; Bolund et al. 2011). Thus, understanding how population-level trait variation has been shaped by selection and how environmental context impacts trait development and expression in an individual can provide insight into the information content of these traits. Additionally, this information about sources of variation is useful for understanding inheritance of traits and how variation is maintained (Miller and Moore 2007). This can be particularly interesting when studying signal traits that are newly developed each year (e.g., plumage in birds, antlers in mammals) or plastic across environmental contexts. While we know relatively little about how the environment influences future signal development, there is evidence that sensitivity to the natal environment has important consequences for an individual's future reproductive success and survival (Merilä and Svensson 1997; Verhulst et al. 1997; Nowicki et al. 2002; Tilgar et al. 2009).

In many oviparous species where development occurs in a discrete nest location, a key aspect of the developmental environment is parental care, which can vary in terms of quality within a species. Additionally, the nest environment – an extension of parental care – itself can vary widely in terms of microclimate, the number of siblings in a nest, nest parasites, and many other factors that may impact the development and the expression of traits later in life (Lindström 1999). For example, in great tits (*Parus major*), nestlings with greater mass are more likely to acquire high-quality breeding habitat as an adult (Verhulst et al. 1997). Traits affected by developmental conditions are not limited to those related to life history and survival; in many insects, conditions during development covary with the expression of secondary sexual ornaments, thus impacting reproductive success (Emlen 1994; Moczek and Emlen 1999; Bonduriansky 2007; Punzalan et al. 2008). Moreover, sexual signals in birds are also known to reflect early environmental conditions. Nutritional status during early development has been shown to influence brain development in birds, which in turn affects song production used in attracting mates and defending territories (Nowicki et al. 1998, 2002). Poor brain development early in life often leads to poor-quality song production as an adult, lowering a males ability to acquire a mate (Buchanan et al. 2003; Spencer et al. 2003; MacDonald et al. 2006).

Early environment is also implicated in causing variation in adult and juvenile plumage coloration. In blue tit nestlings, the development of both structural (UV/blue) and carotenoid-based (yellow) plumage has been shown to be associated with nestling body condition and nest environment (Hadfield and Owens 2006; Hadfield et al. 2007; Johnson and Burnham 2013). The effects of nest environment can also impact adult coloration as demonstrated by quantitative genetic studies of bib size in house sparrows (*Passer domesticus*) (Jensen et al. 2006) and plumage color in great tits (*Parus major*), detectable even after multiple molts (Evans and Sheldon 2012). Experimental manipulation of dietary carotenoids in hihi (*Notymystis cincta*) nestlings influenced the color of adult white ear tufts, but did not impact juvenile or adult carotenoid- or melanin-based colors (Walker et al. 2013). Additionally, longitudinal studies have demonstrated condition and age effects for carotenoid-based (Evans and Sheldon 2013), structural (Siefferman et al. 2005), and melanin-based plumage colors (Bradley et al. 2014; D'Alba et al. 2014), yet little is known about whether juvenile phenotype is indicative of adult phenotype within an individual. Establishing links between natal and adult phenotypes would be extremely useful when studying species in which information on the developmental environment of adults is unknown, such as in many migratory passerines where rates of natal mortality and dispersal are high.

In addition to postnatal developmental conditions, there is extensive evidence that prenatal environmental conditions (maternal effects such as physiological condition during egg development and yolk deposition) can significantly impact an individual's phenotype. For example, recent medical research suggests that, in humans, maternal diet during pregnancy can influence an offspring's predisposition to diabetes and obesity during childhood and adolescence as well as their predisposition to associated diseases in adulthood (Rooney and Ozanne 2011). Maternal effects can include elements of the developmental, or maternal, environment; for example, among females, there will be variation in nest site, brooding time/temperature, and feeding rate that might influence offspring phenotype (Miller and Moore 2007; Räsänen and Kruuk 2007). Maternal effects also include elements of the prenatal environment that vary among mothers such as condition, hormone levels, and behavior (Mousseau and Fox 1998; Räsänen and Kruuk 2007; Tschirren et al. 2012). Variation in maternal phenotype is often due to genetic variation among females, and therefore, variation in offspring phenotype due to maternal phenotype is considered an indirect genetic effect, while variation due to offspring genotype is a direct genetic effect (Miller and Moore 2007). Recently, it has been argued that maternal effects and their influence on offspring phenotype can lead to evolutionary change and are important to consider when studying the evolution of signal traits (Mousseau and Fox 1998; Miller and Moore 2007; Räsänen and Kruuk 2007; Duckworth et al. 2015).

A thorough understanding of the potential mechanisms that underlie signal trait variation involves knowing (1) the relative influence of different sources of phenotypic variation, (2) how a phenotype changes and develops throughout an individual's lifetime, and (3) how different sources of phenotypic variation might interact in the production of a phenotype (Danchin 2013). Miller and Moore (2007) suggest that the expression of sexual signals can often be influenced by a combination of additive genetic effects, nongenetic environmental effects, and indirect genetic effects (additive genetic variation in mothers). By quantifying these different sources of phenotypic variation, we can make predictions about the information content of a signal and the processes that maintain trait variation in and among populations.

In this study, we ask (1) what is the relative influence of genes and the environment – including the prelaying maternal environment – on the expression of phenotypic variation in plumage color, and (2) how does early environment (i.e., nest environment) influence the expression of this trait into adulthood. To address these questions, we quantified the contributions of genetic and environmental variance to the development and expression of a highly variable, continuously distributed melanin-based plumage color trait known to be a target of sexual selection in adult barn swallows (*Hirundo rustica*) (Safran et al. 2005). To explore the relative significance of genetics and the environment on the development of plumage coloration, we leverage naturally occurring variation in extra-pair offspring to compare phenotypes of related individuals raised in the same and in different nests. Further, we compare variation in the color of individuals at different developmental time points to assess the role of early environment, or developmental plasticity, in the production of plumage color.

## Methods

### Study species and study area

Barn swallows have been a model system for sexual selection research for decades (Møller 1994; Turner 2006; Scordato and Safran 2014). Females of the North American subspecies (*H. r. erythrogaster*) do not attend to long tail streamers in males, but rather to darker melanin-based ventral plumage color (McGraw et al. 2004; Safran and McGraw 2004). Coloration is sexually dimorphic and varies within (Safran and McGraw 2004) and between subspecies (Safran and McGraw 2004; Vortman et al. 2011, 2013). Manipulative experiments conducted in two different populations of North American barn swallows have shown that males whose plumage was experimentally enhanced maintain higher paternity in their social broods compared to control males, indicating a causal link between color and reproductive success in multiple North American populations (Safran et al. 2005; R. J. Safran, unpubl. data).

During the 2008 and 2009 breeding seasons, we monitored barn swallows at 24 breeding sites across Boulder, Jefferson, and Weld counties in Colorado that are part of a large, continental population distributed across North America. We attempted to capture all adults at a site using mist nets and targeted night captures. Adults were banded with United States Geological Survey (USGS) aluminum bands and given a unique color combination that consists of randomly chosen plastic color band and/or colored tail spots that allowed for individual identification during behavioral observations. These color combinations do not have an effect on an individual's reproductive success (Spearman's rho, association between number of fledged young and color band: males *ρ* = 0.090, *P* = 0.303, *n* = 133; females *ρ* = −0.113, *P* = 0.137, *n* = 174; tail spot colors: males *ρ* = −0.123, *P* = 0.157, *n* = 133; females *ρ* = −0.061, *P* = 0.425, *n* = 174). We collected morphological measurements (flattened wing length, tail streamer length, and mass), a blood sample from the brachial vein, and a plumage sample from the breast of the bird. As nests were initiated, we identified the social male and female associated with the nest, and documented the clutch initiation date, the clutch size, the hatch date, and the brood size. On day 12 of the nestling period, we banded and measured nestlings (wing length and mass) and took blood and plumage samples. Finally, we estimated when and how many nestlings fledged from successful nests. Despite their socially monogamous mating system, high rates of extra-pair paternity (∼30%) have been reported in many populations of barn swallows (Saino et al. 1997; Safran et al. 2005; Kleven et al. 2006); thus, we determined whether nestlings were within-pair or extra-pair offspring using microsatellite markers (see below).

### Plumage color analyses

Following Safran et al. (2010), feather samples were taped to a standard white card background so that they overlap as they do on the body of a bird. The color of each patch was measured using a spectrometer (USB 4000; Ocean Optics), pulsed xenon light (PX-2; Ocean optics, Dunedin, FL), and SpectraSuite software (v2.0.151). The probe was held perpendicular to the feather surface at a distance such that a 2.5 mm diameter was illuminated and measured. Each sample was measured three times and averaged, with each measurement being an average of 20 scans. From the generated spectra, we quantified color in tetrahedral color space (Stoddard and Prum 2008), a technique that estimates the relative stimulation of the four cone types in songbird eyes (Fig. S1). This technique quantifies color variation in a way that is relevant to the intended receiver. This method yields three metrics that describe hue (theta and phi) and saturation (*r*). Theta and phi are measures of angular displacement that describe where in the three-dimensional color space a particular sample lies. Variation in these hue metrics is due to differences in pigment type and/or nanostructure of the feather; for melanin-based colors, the relative proportion of eumelanin to pheomelanin pigment will influence the values of theta and phi. The third metric, *r*, describes the distance from the achromatic center of the color space (Stoddard and Prum 2008) and will be largely influenced by the concentration of pigment such that feathers with higher pigment concentrations will be more saturated with greater r values. The possible *r* values vary depending on location in the color space of a sample (theta and phi); therefore, here we use r achieved (*r*_A_), which is a measure of r relative to the maximum value of *r* (*r*_max_) possible for that location. We also quantified average brightness of each plumage sample as a measure of how much light, regardless of wavelength, is reflected off the feather surface (Montgomerie 2006); feathers with higher pigment concentration will appear darker as there is more pigment to absorb light. Additionally, brightness and saturation can be influenced by the microstructure of the feather independently of the concentration of pigment making these traits more condition-dependent than theta or phi (D'Alba et al. 2014). Across the repeated measurements for each sample, theta, phi, *r*_A_, and brightness are highly repeatable (*r* = 0.93–0.95; *r* = 0.91–0.93; *r* = 0.82–0.87; *r* = 0.88–0.93, respectively). All plumage color metrics were quantified using the R package pavo (Maia et al. 2013), and repeatability was calculated using the ICC package (Wolak et al. 2012).

We report results for all color metrics (theta, phi, r_A_, and brightness) as variation in these metrics is likely driven by differing genetic and physiological mechanisms relating to melanogenesis (i.e., total amount of pigment vs. proportion of pigment type) (McGraw et al. 2005; McGraw 2006; Hubbard et al. 2010).

### Paternity analyses

DNA samples were extracted from blood taken in the field using Qiagen DNeasy Blood & Tissue Extraction kits (Maryland, USA). Polymerase chain reaction (PCR) was utilized to amplify seven previously developed microsatellite loci – Escu6: (Hanotte et al. 1994); Ltr6: (McDonald and Potts 1994); Pocc6: (Bensch et al. 1997); and Hir11, Hir19, and Hir20: (Tsyusko et al. 2007); and Hru6: (Primmer et al. 1995). Reaction conditions for pooled Escu6, Ltr6, Hir20, and Hir11 primers consisted of 50–100 ng DNA, 0.12 mmol/L of each labeled forward primer, 0.12 mmol/L of each reverse primer, 200 *μ*mol/L each dNTP, 3.25 mmol/L MgCl_2_, 1× PCR buffer, and 0.15 units Taq polymerase (New England Biolabs, MA), and were amplified with the following protocol: initial denaturation step of 94°C for 1 min, followed by 10 cycles of 94°C for 30 sec, 55°C for 30 sec, and 72°C for 45 sec, with an additional 25 cycles starting at 87°C for 30 sec instead of 94°C, and completed with a final extension at 72°C for 3 min. The Pocc6 reaction was modified from the above conditions using 1.25 mmol/L MgCl_2_, and the above conditions were altered for the Hir19 reaction with 3 mmol/L MgCl_2_, and 0.2 mmol/L each forward and reverse primer. The PCR amplification protocol for Pocc6, Hru6, and Hir19 was similar to the previously described protocol for the pooled reaction with the exception that 60°C was used for the annealing temperature. Amplified PCR products containing the fluorescently labeled forward primer were detected using an ABI3730 DNA analyzer (Applied Biosystems, Grand Island, NY).

Genotypes for nestlings and adults were assigned using GeneMapper software (v4.0, Applied Biosystems, Grand Island, NY). Genotypes from adults and offspring were incorporated into a paternity analysis using CERVUS software (v2.0) to calculate exclusion probabilities and assign paternities. Paternity exclusion was conducted using similar parameters described in Neuman et al. (2007). Briefly, we considered young as extra-pair if we detected two or more alleles (from the seven microsatellite loci) that did not match the social father.

### What is the relative influence of genes and environment on juvenile color?

To examine the quantitative genetics of plumage color, we used the animal model, a mixed-effects model that partitions phenotypic variance into different components, such as environmental, genetic, and maternal effects, from which heritability and other parameters can be estimated (Lynch and Walsh 1998; Kruuk 2004; Wilson et al. 2010). This analytical tool has traditionally been used in animal breeding where pedigrees are closely monitored. With the increasing ease of molecular paternity analyses, availability of long-term datasets, more affordable computing power, and access to user-friendly analytical software, it has become a popular tool for natural systems. We estimated the variance components for each color metric by fitting a multivariate animal model using a Bayesian Markov chain Monte Carlo (MCMC) technique implemented in the R package MCMCglmm (Hadfield 2010). To estimate additive genetic variance, the animal model generates a relatedness matrix from a pedigree. Our pedigree consisted of 511 offspring with 108 mothers and 95 fathers with one individual represented as both offspring and mother for a total of 713 identities total. We do not have any information regarding the relatedness of the mothers and fathers in this pedigree; thus, we assumed that breeding adults are unrelated. We feel confident in this assumption as individuals recruited into the population are rarely related.

For the following analyses, we standardized all numerical variables to their mean following Lande and Arnold (1983). In the model, we initially included year, sampling date (nested within year), nestling mass, and nestling sex as fixed effects; only year had a statistically significant effect on the model; therefore, we excluded the other fixed effects in our final model (Wilson et al. 2010). To partition total phenotypic variance into additive genetic variance and nest environmental variance, we included the following random effects: (1) pedigree and (2) nest identity. Total phenotypic variance (*V*_P_) of each color metric was calculated as the sum of the variance components: *V*_P_ = *V*_A_ + *V*_CE_ + *V*_R_, where *V*_A_ is the additive genetic variance, *V*_CE_ is the nest environmental variance, and *V*_R_ is the residual variance (see Table 1) (Falconer and Mackay 1996; Lynch and Walsh 1998). Using the variance components, we calculated narrow sense heritability (*h*^2^ = *V*_A_/*V*_P_) and the effect of nest environment (ce^2^ = *V*_CE_/*V*_P_). An advantage of the Bayesian framework used here is that the uncertainty associated with each component carries over into the subsequent variance ratio estimates allowing for Bayesian credible intervals (BCI) to be estimated for heritability and nest environmental effects for each color metric. Because there are significant phenotypic correlations among color metrics within individual nestlings (Table 2), we also estimated the genetic correlation among all pairwise combinations of the four color metrics of the breast plumage.

**Table 1:** Posterior modes of variance components and variance ratio estimates (with 95% BCI) for each color metric estimated from a multivariate animal model (DIC = 3957.982). Variance ratios were calculated as follows: narrow sense heritability (*h*^2^ = *V*_A_/*V*_P_) and nest environment (ce^2^ = *V*_CE_/*V*_P_). Theta and phi are measures of hue, *r*_A_ is a measure of saturation, and brightness is a measure of reflected light

All families					
Estimate		Hue (theta) (95% BCI)	Hue (phi) (95% BCI)	Saturation (*r*_A_) (95% BCI)	Brightness (95% BCI)
*V*_A_	Additive genetic variance	0.34 (0.145–0.687)	0.297 (0.099–0.591)	0.332 (0.106–0.585)	0.297 (0.129–0.648)
*h*^*2*^	*V*_A_/*V*_P_ proportion of total phenotypic variance explained by additive genetic variance	0.346 (0.136–0.605)	0.211 (0.092–0.511)	0.269 (0.126–0.546)	0.312 (0.127–0.584)
*V*_CE_	Nest environment variance	0.303 (0.159–0.462)	0.304 (0.175–0.464)	0.177 (0.094–0.332)	0.293 (0.121–0.416)
ce^2^	*V*_CE_/*V*_P_ proportion of total phenotypic variance explained by nest environment	0.286 (0.164–0.403)	0.28 (0.18–0.404)	0.178 (0.095–0.306)	0.24 (0.124–0.365)
*V*_R_	Residual variance	0.393 (0.183–0.552)	0.459 (0.261–0.616)	0.512 (0.336–0.671)	0.489 (0.253–0.618)
*V*_P_	Total phenotypic variance	1.09 (0.939–1.275)	1.092 (0.943–1.262)	1.055 (0.902–1.193)	1.077 (0.928–1.244)

**Table 2:** Comparison of within-individual phenotypic correlations (below diagonal) and genetic correlations (above the diagonal) among all pairwise combinations of the four color metrics. Significant correlations are in bold

	Hue (theta)	Hue (phi)	Saturation (r_A_)	Brightness
Hue (theta)	–	−**0.699** (−0.883 to −0.316)	−**0.691** (−0.872 to −0.233)	**0.705** (0.240 to 0.870)
Hue (phi)	−**0.706** (−0.747 to −0.659)	–	0.308 (−0.253 to 0.755)	−**0.689** (−0.852 to −0.109)
Saturation (*r*_A_)	−**0.572** (−0.628 to −0.511)	**0.277** (0.195 to 0.355)	–	−**0.778** (−0.916 to −0.399)
Brightness	**0.585** (0.523 to 0.640)	−**0.515** (−0.576 to −0.448)	−**0.748** (−0.784 to −0.708)	–

We also used a subset of our data for which females had multiple broods within or across breeding seasons allowing us to assess maternal effects separate from nest environment. We used this subset as only a small sample of females had multiple broods (*n* = 32) and maternal effects would likely be confounded by environmental effects in the larger dataset. This pedigree consisted of 246 offspring, 32 mothers, and 45 fathers for a total of 323 identities. As with the complete dataset, we first included year, sampling date (nested within year), nestling mass, and nestling sex as fixed effects; however, these did not have a statistical effect on the model and were therefore not included in the final model (Table 3). In addition to pedigree and nest identity, we included maternal identity as random effects to estimate maternal effects. Maternal effects were calculated in the same manner as heritability and the effect of nest environment (me^2^ = *V*_ME_/*V*_P_), where *V*_P_ = *V*_A_ + *V*_CE_ + *V*_ME_ + *V*_R_.

**Table 3:** Posterior mode (and 95% BCI) of all fixed effects included in maximal model (DIC: all families = 3968.275; multiple broods= 1809.114). Only year in the model with all families was significant

	All families (95% BCI)	Females with multiple broods (95% BCI)
Year	**0.082** (0.005–0.135)	0.054 (−0.027–0.199)
Date in 2008	0.026 (−0.015–0.041)	0.015 (−0.034–0.058)
Date in 2009	−0.020 (−0.070–0.043)	−0.026 (−0.143–0.071)
Sex	−0.029 (−0.062–0.008)	−0.034 (−0.085–0.016)
Body mass	0.013 (−0.010–0.036)	0.023 (−0.016–0.058)

Significant fixed effects are indicated in bold.

We specified the priors for variance–covariance matrix as an inverse Wishart matrix distribution (Wilson et al. 2010; De Villemereuil 2012). Final models were run for 1,002,000 iterations, with a burn-in of 2000 iterations, and every 500th iteration was stored (autocorrelations were weaker than 0.048 for all variance components) with effective sample sizes between 1508 and 2000. We varied the priors specified in the final model by adjusting the inverse gamma and beta distributions for variances and correlation and adjusting the number of iterations such that effective sample sizes were similar; the model outcomes were relatively insensitive to prior parameterization (Table S1).

### Does juvenile coloration predict adult signal variation?

**Figure 1:**
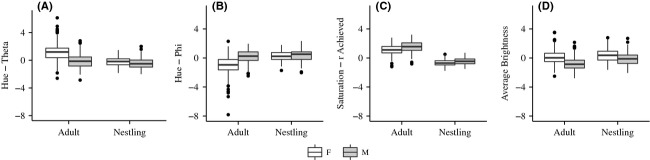
Color differences between developmental stages and sexes (females in white, males in gray) – (A) shows difference for theta (a measure of hue); (B) shows differences in phi (a measure of hue); (C) shows differences in r achieved (a measure of color saturation); and (D) shows differences in achromatic brightness. In both nestlings and adults, males are significantly darker (lower average brightness) with more saturated color (higher *r* achieved). The two measures of hue (theta and phi) are dimorphic in adults, but not nestlings.

As in many migratory passerines, recruitment of juvenile barn swallows into their natal population is low (approximately 1% in our study population) and comparisons of adult color between individuals of known relatedness are therefore extremely difficult. However, nestlings begin to grow juvenile feathers with qualitatively similar coloration to that of adults, while they are still in the nest (Fig. 1). Since we began monitoring this population in 2008, a small number of individuals banded and sampled as nestlings have returned as breeding adults in their first year (total for which we have plumage samples at both time points through 2013: *n* = 76; males = 56 and females = 20); these individuals are representative of all nestlings banded in our study population in terms of ventral color. Using these individuals, we modeled the linear relationship between color measured within the same individual at two different stages (standardized to the mean): as a nestling and as an adult in their first breeding season to determine if juvenile plumage color predicted adult color within individuals. We included sex as a covariate in this model as color is dimorphic in this subspecies (Safran and McGraw 2004). Barn swallows go through their first basic molt on the wintering grounds before their first breeding season; consequently, the plumage samples taken from adults in their first breeding season were grown at a very different time and place than plumage samples taken from juvenile birds; thus, any within-individual similarity in coloration cannot be due to plumage color being developed in the same environment. All statistical analyses were performed in R v3.0.3 (R Core Team, 2014).

## Results

### Relative influence of genes and environment on juvenile plumage color expression

#### Using mixed paternity broods to analyze the influence of genes and the environment on trait expression

**Figure 2:**
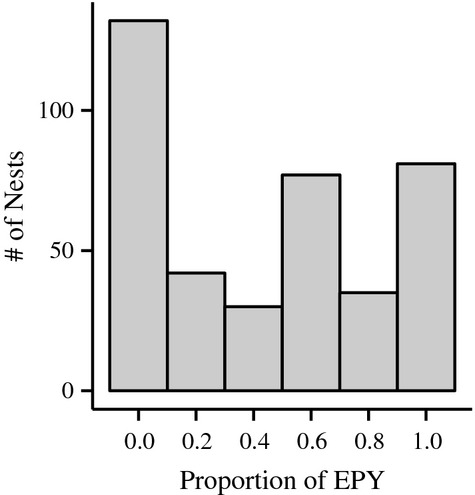
Histogram showing the proportion of extra-pair young (EPY) in a nest; data pooled across all breeding seasons from 2008 to 2012.

We assigned genotypes for 512 nestlings and 125 parental pairs for all seven loci. With a combined first-parent exclusion probability of 99.88% for all seven loci, we were able to assign 303 nestlings as within-pair young (sired by social father) and 209 (40.8%) as extra-pair young (not sired by social father). Of those 209 extra-pair young, we were able to determine the identity of the extra-pair father for 63 nestlings. Based on all families genotyped between 2008 and 2012, we found a similarly high rate of extra-pair paternity in our study population, with, on average, 41.1% of nestlings sired by extra-pair males. On average, 66.8% of nests contained at least one extra-pair young (EPY), with some nests having 100% EPY (see Table 4 for annual percentages; Fig. 2).

**Table 4:** Summary of annual extra-pair young (EPY) rates. Both total percent of young that are EPY and percent of nests with EPY are shown

	% of EPY in population	% of nests with EPY
2008	44.31	65.93
2009	36.60	64.23
2010	40.00	60.53
2011	36.36	64.29
2012	45.88	76.71
All years	41.07	66.75

The model to partition phenotypic variance into additive genetic variance and nest environmental variance included pedigree and nest identity as random effects (Table 1). From these variance components, we calculated variance ratios to determine the relative effect of shared genes (h^2^) and shared nest environment (ce^2^) for each color descriptor (Table 1).

#### Additive genetic variation

The high occurrence of EPY in our populations created a half-sib/full-sib structure among offspring. In this population, additive genetic variance (h^2^) explains approximately 28% of the phenotypic variation in melanin-based plumage coloration (Table 1). Additionally, within-individual phenotypic correlations for each color metric was reflected at the genetic level as we found strong genetic correlations for each pairwise combination of color metrics (Table 2); phi and r_a_ showed the weakest phenotypic correlation, and the genetic correlation for these two measures of color was not significant.

#### Nest environment

While most related individuals also shared the same nest environment, our dataset consisted of several maternal and paternal half siblings that experienced different environments. We determined that nest environment (ce^2^) also explains a significant proportion (approximately 25%) of phenotypic variation in coloration (Table 1). Combined, additive genetics (h^2^) and nest environment (ce^2^) explained 47% of phenotypic variation.

#### Maternal effects

To estimate the influence of maternal identity on color variation, we used a subset of the data for which mothers had multiple broods, the model included pedigree, nest identity, and maternal identity as random effects to partition phenotypic variance into additive genetic variance, nest environmental variance, and prelaying maternal environmental variance (Table 5). From these variance components, we calculated variance ratios to determine the relative influence of additive genetics (h^2^), nest environment (ce^2^), and maternal effects (me^2^) for each color descriptor (Table 5).

**Table 5:** Posterior modes of variance components and variance ratio estimates (with 95% BCI) for each color metric from a multivariate animal model using only females that had multiple broods within or across years allowing phenotypic variance due to the prelaying maternal environment to be estimated (DIC = 1806.195). Variance ratios were calculated as follows: narrow sense heritability (*h*^2^ = *V*_A_/*V*_P_), nest environment (ce^2^ = *V*_CE_/*V*_P_), and prelaying maternal environment (me^2^ = *V*_ME_/*V*_P_)

Females with multiple broods					
Estimate		Hue (theta) (95% BCI)	Hue (phi) (95% BCI)	Saturation (*r*_A_) (95% BCI)	Brightness (95% BCI)
*V*_A_	Additive genetic variance	0.256 (0.101–0.742)	0.28 (0.083–0.748)	0.266 (0.088–0.627)	0.186 (0.084–0.637)
*h*^*2*^	*V*_A_/*V*_P_ proportion of total phenotypic variance explained by additive genetic variance	0.197 (0.068–0.512)	0.203 (0.08–0.508)	0.198 (0.072–0.459)	0.18 (0.058–0.457)
*V*_CE_	Nest environment variance	0.361 (0.157–0.62)	0.274 (0.133–0.572)	0.268 (0.112–0.475)	0.217 (0.117–0.465)
ce^*2*^	*V*_CE_/*V*_P_ proportion of total phenotypic variance explained by nest environment	0.254 (0.121–0.399)	0.224 (0.121–0.383)	0.178 (0.105–0.344)	0.186 (0.098–0.333)
*V*_ME_	Maternal environment variance	0.135 (0.067–0.374)	0.14 (0.065–0.368)	0.141 (0.066–0.398)	0.145 (0.06–0.386)
me^2^	*V*_ME_/*V*_P_ proportion of total phenotypic variance explained by maternal environment	0.119 (0.058–0.255)	0.091 (0.055–0.243)	0.155 (0.061–0.276)	0.135 (0.057–0.27)
*V*_R_	Residual variance	0.452 (0.165–0.656)	0.493 (0.203–0.706)	0.453 (0.263–0.718)	0.523 (0.288–0.743)
*V*_P_	Total phenotypic variance	1.343 (1.053–1.697)	1.343 (1.072–1.721)	1.233 (1.02–1.629)	1.272 (1.053–1.625)

When maternal identity is included in the model, the phenotypic variation explained by both additive genetics (h^2^) and nest environment (ce^2^) decreased (approximately 19% and 21%, respectively); phenotypic variation in coloration explained by prelaying maternal environment was approximately 13% (Table 5). Combined, additive genetics (h^2^), nest environment (ce^2^), and maternal effects (me^2^) explained 47% of phenotypic variation.

### Juvenile coloration predicts adult signal

#### Differences between nestlings and adults

Using color data from adults and nestlings sampled in a breeding season when no manipulative experiments were conducted (2011), we explored age and sex differences in plumage color using two-way ANOVAs for each color metric (theta, phi, *r*_A_, and average brightness). In each model, the interaction between sex and age was significant (theta: *F*_1, 611_ = 38.89, *P* < 0.0001; phi: *F*_1, 611_ = 38.28, *P* < 0.0001; *r*_A_: *F*_1, 611_ = 2.39, *P* < 0.013; and brightness: *F*_1, 611_ = 4.43, *P* = 0.023), and we used a Tukey's post hoc analysis to assess the pairwise comparisons of interest. In adults, we found significant sexual dichromatism in all four color metrics; on average, males are darker with more saturated color (higher *r*_A_) and significant differences in theta and phi suggest that males and females occupy different color space (Fig. 1; Table 6). We also found that nestlings are significantly dichromatic with differences in r_A_ and brightness that mirror those found in adults; however, there are no significant differences in theta and phi in nestlings (Fig. 1; Table 6). Nestlings also significantly differ in color from adults; in particular, adults have more saturated color (*r*_A_) relative to nestlings (Fig. 1; Table 6).

**Table 6:** Pairwise differences (with 95% CI) from Tukey's post hoc analysis comparing color among sex and developmental stages. Significant differences are in bold

	Hue (theta)	Hue (phi)	Saturation (*r*_A_)	Brightness
	Males versus females	Males versus females	Males versus females	Males versus females
Nestlings	−0.209 (−0.545 to 0.127)	0.160 (−0.180 to 0.500)	**0.209** (0.003 to 0.414)	−**0.497** (−0.801 to −0.192)
Adults	−**1.251** (−1.525 to −0.978)	**1.181** (0.905 to 1.458)	**0.464** (0.297 to 0.631)	−**0.844** (−1.091 to −0.596)

	Nestlings versus adults	Nestlings versus adults	Nestlings versus adults	Nestlings versus adults
Males	−**0.327** (−0.626 to −0028)	0.212 (−0.090 to 0.500)	−**2.005** (−2.188 to −1.823)	**0.596** (0.325 to 0.867)
Females	−**1.369** (−1.683 to −1.056)	**1.233** (0.916–1.550)	−**1.750** (−1.942 to −1.559)	0.249 (−0.035 to 0.533)

#### Nonrandom recruitment of nestlings as breeders?

**Figure 3:**
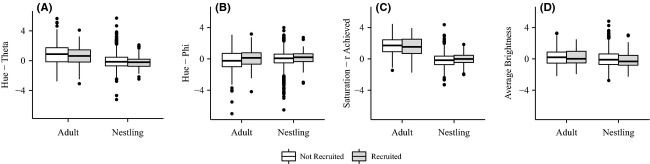
Color differences between individuals recruited into their natal population and individuals that dispersed to/from another population. White boxes represent adults that were recruited from a different population or nestlings that dispersed or did not survive. The gray boxes represent adults recruited into their natal population or nestlings that returned as breeding adults. (A) Shows difference for theta (a measure of hue); (B) Shows differences in phi (a measure of hue); (C) Shows differences in r achieved (a measure of color saturation); and (D) Shows differences in achromatic brightness.

For the color comparisons between recruited and nonrecruited individuals, we used Welch's two-sample *t*-test, which assumes unequal variance in groups. This analysis is appropriate as our samples sizes for the two groups were unequal (returning nestlings = 84; nonreturning nestlings = 3466; adults recruited from natal population = 79; adults recruited from a different population = 267; sample sizes for recruited individuals differ due to missing color data at one time point) leading to unequal variances for each pairwise comparison. We found no statistically significant difference in plumage coloration between returning nestlings compared to nonreturning nestlings from the same years (theta: *t*_87.01_ = −1.04, *P* = 0.30; phi: *t*_87.57_ = 1.33, *P* = 0.19; *r*_A_: *t*_87.08_ = 1.65, *P* = 0.10; brightness: *t*_86.33_ = −0.75, *P* = 0.45); therefore, we infer that the returning nestlings are a representative subset of the nestlings hatched in our population. As adults, individuals hatched in our study area and recruited into the breeding population generally did not differ in plumage color compared to first-time breeding adults recruited from a different population (theta: *t*_138.04_ = −1.00, *P* = 0.32; *r*_A_: *t*_108.47_ = −1.18, *P* = 0.24; brightness: *t*_131.22_ = −0.46, *P* = 0.65). While there was a statistically significant difference in one of the metrics of hue, phi (*t*_145.18_ = 2.23, *P* = 0.027); Figure3 shows that the distributions of phi for adults recruited from their natal population and those recruited from other populations overlap.

#### Longitudinal analyses: predicting color across years and life stages

**Figure 4:**
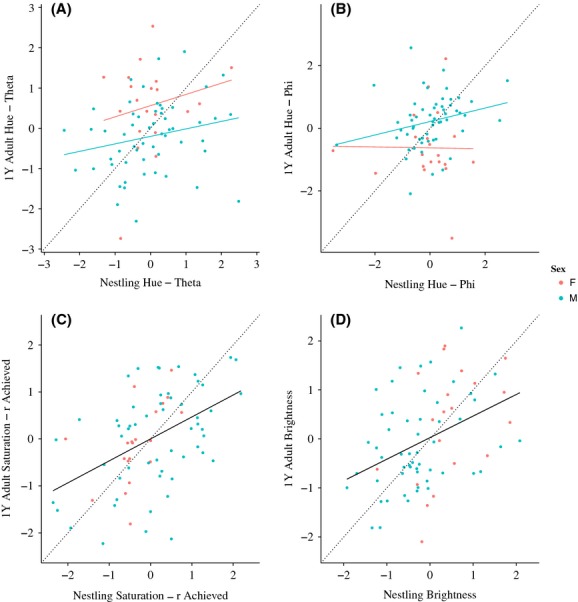
Within-individual color is predictive from one developmental stage to the next. Results of ANCOVAs demonstrate that the y-intercept for males and females is significantly different, but not the slope for theta (A) and phi (B), and there is no differences between males and females for *r*_A_ (C) and brightness (D) ((A)theta – *b* = 0.20, *t* = 1.87, *P* = 0.07, ANCOVA – *F*_2, 73_ = 6.70, *R*^2^ = 0.13, *P* = 0.002; (B) phi – *b* = 0.15, *t* = 1.38, *P* = 0.17, ANCOVA – *F*_2, 73_ = 7.08, *R*^2^ = 0.14, *P* = 0.002; (C) *r*_A_ – *b* = 0.47, *t* = 4.55, *P* < 0.0001, ANCOVA – *F*_1, 74_ = 20.72, *R*^2^ = 0.21, *P* < 0.0001; (D) average brightness – *b* = 0.36, *t* = 3.35, *P* = 0.001, ANCOVA – *F*_1, 74_ = 11.22, *R*^2^ = 0.12, *P* = 0.001).

Within an individual, 12-day-old nestling plumage color significantly predicted adult plumage color in their first breeding season. We found that regardless of sex, there is a significant relationship between nestling and adult plumage for *r*_A_ (*b* = 0.47, *t* = 4.55, *P* < 0.0001, ANCOVA – *F*_1, 74_ = 20.72, *R*^2^ = 0.21, *P* < 0.0001, Fig. 4C) and average brightness (*b* = 0.36, *t* = 3.35, *P* = 0.001, ANCOVA – *F*_1, 74_ = 11.22, *R*^2^ = 0.12, *P* = 0.001, Fig. 4D). We also found a positive trend between nestling and adult color for measures of hue; while sex significantly impacted the intercept, there was no effect on the slope (theta – *b* = 0.20, *t* = 1.87, *P* = 0.07, ANCOVA – *F*_2, 73_ = 6.70, *R*^2^ = 0.13, *P* = 0.002, Fig. 4A; phi – *b* = 0.15, *t* = 1.38, *P* = 0.17, ANCOVA – *F*_2, 73_ = 7.08, *R*^2^ = 0.14, *P* = 0.002, Fig. 4B). The finding that nestling color predicts adult color, and that nestling color is significantly influenced by nest environment, indicates that early environment has lasting effects on future plumage color development in barn swallows.

## Discussion

In barn swallows, variation in plumage color is predictive of individual reproductive performance (Safran and McGraw 2004; Safran et al. 2005). Here, we report that juvenile plumage color is affected by both nest environment and maternal effects and that juvenile plumage color predicts an individual's plumage color as a first-time breeding adult. Taken together, these results indicate that the environment an individual experiences as a developing nestling has long-term effects on sexual signal development and therefore on reproductive performance.

### Sources of phenotypic variation

Using the animal model, total phenotypic variance for each color metric (theta, phi, *r*_A_, and average brightness) was partitioned into three variance components: additive genetics, nest environment, and prelaying maternal environment (Table 1). Previous work on melanin-based coloration in vertebrates has revealed that variation in polymorphic colors (e.g., the black and tan colors of rock pocket mice) can be largely explained by underlying genotypes (Hoekstra et al. 2004; Mundy 2005; Uy et al. 2009; Hubbard et al. 2010). However, here we find that the effect of additive genetics (*h*^2^) is relatively low for a melanin-based color trait that varies from light to dark on a continuous scale (see Roulin and Dijkstra 2003; Griffith et al. 2006; Saino et al. 2013). Alternatively, nest and prelaying maternal environment combined explain the largest proportion of total phenotypic variation in barn swallow coloration (Table 5). Collectively, these results suggest that offspring receiving common alleles will produce somewhat similar phenotypes; however, individuals that experience the same nest and prelaying maternal environments will produce highly similar phenotypes. Consequently, females might differentially allocate paternity to males with darker, more saturated plumage color (Safran et al. 2005) because it provides information about his developmental conditions, which may directly affect a male's parental quality or indicate that he possesses “good” maternal care alleles that will be passed on to daughters (Miller and Moore 2007). Alternatively, females might choose a nesting site based on environmental conditions as previous results indicate that competition among males for the best nests results in the pattern that darker, more saturated males begin breeding earlier than their duller counterparts (Safran and McGraw 2004). In either scenario, we would predict that darker males would sire darker sons; however, an experiment that decouples nest quality from male color is needed to differentiate between these two hypotheses.

### Heritability of melanin-based color

In contrast to other studies that estimate heritability of melanin-based plumage color (Grant 1990; Mundy 2006; Potti and Montalvo 2008; Saino et al. 2013), our study reveals fairly low – though nontrivial – degree of heritability for plumage color in North American barn swallows (*h*^*2*^ ≅ 0.28). In a different population of barn swallows Saino et al. (2013) found that ventral plumage color was highly heritable (*h*^*2*^ ≅ 0.80) using parent–offspring regressions. However, analyses such as parent–offspring regression that do not control for other sources of nonindependence such as shared environment may overestimate heritability. Consequently, the animal model approach used here allows for a more accurate partitioning of phenotypic variance (Kruuk 2004; Wilson et al. 2010). Moreover, in this study, we exploited a natural half-sib/full-sib structure created by a high rate of extra-pair young (∼41%) that allowed us to estimate the effects of genetic and environmental variation. However, in our study system, related individuals are likely to experience the same nest and prelaying maternal environments; consequently, our estimates for heritability and developmental environmental effects may be confounded such that phenotypic variation due to additive genetic variation is being attributed to environmental variation, or vice versa. Future work to experimentally isolate genetic and environmental effects (i.e., cross-fostering experiments) will be quite illuminating (Lindström 1999).

### Developmental Plasticity

Our study also demonstrates that within an individual, plumage color during development is predictive of plumage color as a first-time breeding adult. This is particularly interesting given that plumage color is developed several times within an individual's lifetime: first, in the natal environment on breeding grounds in North America and subsequently, once per year during the nonbreeding season in Central and South America before they migrate back to breeding sites. If an individual's underlying genotype explained this pattern of within-individual variation, we would expect much higher heritability estimates with related individuals having highly similar phenotypes as they are more likely to have the same underlying genotype. Therefore, we infer there is developmental plasticity for melanin-based plumage color as a function of the nest environment, and this plasticity has long-term effects such that nest environment influences the adult phenotype despite a subsequent molt after leaving the nest environment.

In birds, developmental conditions have been shown to affect many aspects of an individual's phenotype and fitness, including survival (Merilä and Svensson 1997), future clutch size (Haywood and Perrins 1992), and the ability to obtain and defend high-quality breeding habitat (Verhulst et al. 1997). Additionally, early conditions can have significant impacts on important sexual signals such as song (Nowicki et al. 1998), plumage traits (Scordato et al. 2012), and morphology (Ohlsson et al. 2002). Here, we show that the environmental conditions experienced by a nestling barn swallow during the first few weeks of life affect the development of a colorful sexual signal known to affect reproductive success (Safran et al. 2005).

### Maternal effects

This study additionally revealed that variation in plumage color was largely explained by the prelaying maternal environment, which differs from the nest environment as related offspring raised in different nesting attempts within and across breeding seasons will experience different nest conditions, but are likely to experience the same prelaying maternal environment. For example, a female's condition and phenotype can influence hormone deposition in eggs, which is known to vary among female barn swallows (Safran et al. 2008), as well as other passerines (Groothuis and von Engelhardt 2005; Müller et al. 2012). However, in this study, it is impossible to differentiate between prenatal effects such as hormone deposition and postnatal behavioral effects such as parental care. Consequently, the decrease in variation explained by nest environment when prelaying maternal environment is included in the model may be the result of similar parental care behaviors. An experiment where individuals experience the same prelaying maternal environment, but are raised by unrelated females (or parent pairs) would help clarify how maternal effects influence color expression in barn swallows (White et al. 1968; Beamonte-Barrientos et al. 2010).

### Early environment impacts a sexually selected trait

A causal relationship between color and paternity exists in the two studied populations of North American barn swallows, such that darker males are allocated more paternity by their social mate (Safran et al. 2005; Safran, unpubl. data). Plumage color is unique in that it is redeveloped annually and consequently subject to environmental influences during regrowth. Results from this study suggest that rather than signaling how a male is impacted by the current (or recent) environmental context, male color, particularly saturation (*r*_A_), and brightness (D'Alba et al. 2014) may provide information about conditions, including maternal effects, an individual experienced during early development. Moreover, developmental conditions may impact other behavioral, physiological, and morphological traits that affect female mate choice. For example, empirical support for the nutritional stress hypothesis (Nowicki et al. 1998, 2002) shows that early developmental conditions have drastic effects on song learning and production later in life in many species of songbirds. As song is often the target of sexual selection via mate choice, the nutritional stress hypothesis may provide a mechanism for maintaining the reliability of a key sexual signal; a similar mechanism (early developmental conditions) could maintain the reliability of plumage color in barn swallows.

## Conclusion

Ventral plumage coloration in North American barn swallows is representative of many melanin-based color traits with continuous variation. Results from this study demonstrate that both the underlying genes and the environment in which feathers are developed influence juvenile plumage color. Moreover, early environment during development (through maternal effects and features of the nest environment) has lasting effects on adult phenotype, a pattern that has not been well documented for melanin-based plumage color (but see Griffith et al. 1999). Ventral plumage coloration in adult male swallows is known to impact reproductive success in terms of differential paternity allocation by mates (Safran et al. 2005); consequently, early nest environment likely has long-term effects on an individual's lifetime fitness. Given this link between sexually selected plumage color and an individual's developmental environment, females may use this trait in mate choice decisions because it can convey information about the early developmental conditions a male has experienced as well as the maternal care alleles he possesses (Miller and Moore 2007). Moreover, because of the influence of the early environment on signal development, the nest site may be an important feature of mate choice in this system. Future work aimed at more finely disentangling the roles of genetic and environmental variation on the development of this trait via cross-fostering experiments will enable researchers to causally isolate the effects of these two sources of variation and identify specific environmental factors impacting signal development.

## Supporting Information

**Figure S1.** Representation of barn swallow plumage color in tetrahedral color space and visualization of the three color metrics (Stoddard and Prum 2008; Maia et al. 2013).

**Table S1.** Summary of model outcomes using various priors; Final model – inverse gamma and beta = 1, 1 – inverse gamma and beta = 0.5, 2 – inverse gamma and beta = 0.1.
