# Supplementary material for: Quantitative genetics of plumage color: lifetime effects of early nest environment on a colorful sexual signal
Source: Ecol Evol. 2015 Jul 24;5(16):3436–49. doi: 10.1002/ece3.1602 (PMC4569038; doi:10.1002/ece3.1602)

Figure S1. Representation of barn swallow plumage color in tetrahedral color space and visualization of the three color metrics (Stoddard and Prum, 2008; Maia *et al.*, 2013). A) Random sample of 50 adults showing four plumage patches – throat in green, breast in red, belly in blue, vent in yellow; B) only adult breast feathers of the 50 random adults, C) random sample of 50 nestlings for which we are only able to measure breast feathers.

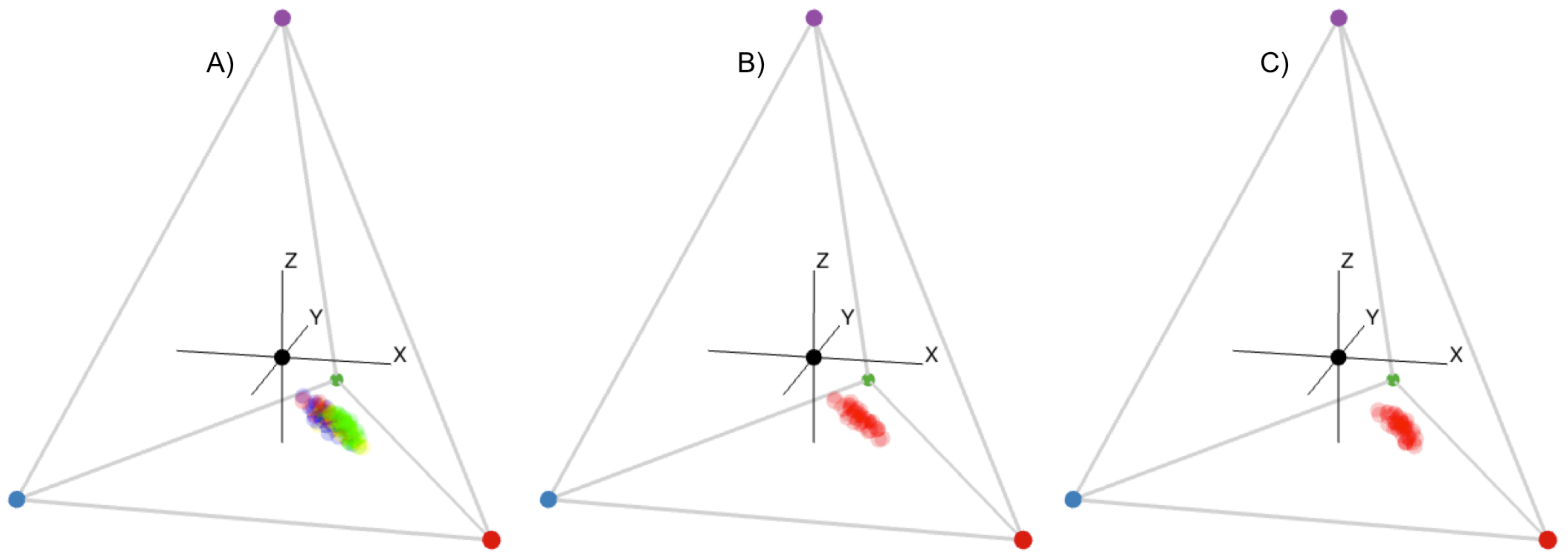

Supplement: Supplementary file 1 — Figure S1. Representation of barn swallow plumage color in tetrahedral color space and visualization of the three color metrics (Stoddard and Prum 2008; Maia et al. 2013). [file ece30005-3436-sd1.pdf]
