# Supplementary material for: Quantitative genetics of plumage color: lifetime effects of early nest environment on a colorful sexual signal
Source: Ecol Evol. 2015 Jul 24;5(16):3436–49. doi: 10.1002/ece3.1602 (PMC4569038; doi:10.1002/ece3.1602)
Supplement: Supplementary file 2 — Table S1. Summary of model outcomes using various priors; Final model – inverse gamma and beta = 1, 1 – inverse gamma and beta = 0.5, 2 – inverse gamma and beta = 0.1. [file ece30005-3436-sd2.docx]

**Table S1.** Summary of model outcomes using various priors; Final model - inverse gamma and beta = 1, 1 - inverse gamma and beta = 0.5, 2 - inverse gamma and beta = 0.1. model outcomes were relatively insensitive to the minor changes in prior parameterization. However, inverse gamma and beta distributions set to 0.1 or lower are not well defined and can cause instability in the MCMC (de Villemereuil, 2012). The DIC and average effective sample size for variance components are as follows: Final – DIC = 3957.982, n = 1894.741; 1 – DIC = 3909.146, n = 1860.006, 2 – DIC = 3387.223, n = 1432.645.

|  | **Theta** | | | **Phi** | | | **r Achieved** | | | **Brightness** | | |
| --- | --- | --- | --- | --- | --- | --- | --- | --- | --- | --- | --- | --- |
|  | **final** | **1** | **2** | **final** | **1** | **2** | **final** | **1** | **2** | **final** | **1** | **2** |
| **V_A_** | 0.340 | 0.402 | 0.578 | 0.297 | 0.192 | 0.189 | 0.332 | 0.279 | 0.239 | 0.297 | 0.333 | 0.361 |
|  | (0.145 - 0.687) | (0.101 - 0.709) | (0.154 - 0.967) | (0.099 - 0.591) | (0.057 - 0.575) | (0.017 - 0.659) | (0.106 - 0.585) | (0.081 - 0.593) | (0.062 - 0.690) | (0.129 - 0.648) | (0.094 - 0.686) | (0.050 - 0.845) |
|  |  |  |  |  |  |  |  |  |  |  |  |  |
| **h^2^** | 0.346 | 0.395 | 0.540 | 0.211 | 0.199 | 0.242 | 0.269 | 0.262 | 0.410 | 0.312 | 0.372 | 0.377 |
|  | (0.136 - 0.605) | (0.114 - 0.631) | (0.159 - 0.818) | (0.092 - 0.511) | (0.061 - 0.504) | (0.016 - 0.567) | (0.126 - 0.546) | (0.077 - 0.548) | (0.061 - 0.630) | (0.127 - 0.584) | (0.087 - 0.606) | (0.042 - 0.730) |
|  |  |  |  |  |  |  |  |  |  |  |  |  |
| **V_E_** | 0.303 | 0.299 | 0.221 | 0.304 | 0.274 | 0.320 | 0.177 | 0.163 | 0.114 | 0.293 | 0.206 | 0.160 |
|  | (0.159 - 0.462) | (0.152 - 0.473) | (0.101 - 0.421) | (0.175 - 0.464) | (0.172 - 0.479) | (0.156 - 0.469) | (0.094 - 0.332) | (0.065 - 0.300) | (0.031 - 0.292) | (0.121 - 0.416) | (0.107 - 0.424) | (0.060 - 0.390) |
|  |  |  |  |  |  |  |  |  |  |  |  |  |
| **e^2^** | 0.286 | 0.230 | 0.196 | 0.280 | 0.282 | 0.275 | 0.178 | 0.184 | 0.115 | 0.240 | 0.235 | 0.183 |
|  | (0.164 - 0.403) | (0.143 - 0.402) | (0.098 - 0.371) | (0.180 - 0.404) | (0.171 - 0.407) | (0.154 - 0.410) | (0.095 - 0.306) | (0.074 - 0.288) | (0.031 - 0.278) | (0.124 - 0.365) | (0.098 - 0.368) | (0.066 - 0.363) |
|  |  |  |  |  |  |  |  |  |  |  |  |  |
| **V_R_** | 0.393 | 0.415 | 0.299 | 0.459 | 0.494 | 0.461 | 0.512 | 0.548 | 0.508 | 0.489 | 0.490 | 0.420 |
|  | (0.183 - 0.552) | (0.185 - 0.582) | (0.054 - 0.555) | (0.261 - 0.616) | (0.277 - 0.647) | (0.226 - 0.694) | (0.336 - 0.671) | (0.322 - 0.696) | (0.270 - 0.706) | (0.253 - 0.618) | (0.233 - 0.637) | (0.158 - 0.658) |
|  |  |  |  |  |  |  |  |  |  |  |  |  |
| **V_P_** | 1.090 | 1.067 | 1.112 | 1.092 | 1.044 | 1.059 | 1.055 | 1.017 | 1.015 | 1.077 | 1.057 | 1.081 |
|  | (0.939 - 1.275) | (0.931 - 1.270) | (0.932 - 1.300) | (0.943 - 1.262) | (0.924 - 1.258) | (0.907 - 1.245) | (0.902 - 1.193) | (0.884 - 1.165) | (0.881 - 1.164) | (0.928 - 1.244) | (0.927 - 1.245) | (0.924 - 1.253) |
